# Assessment of the Preparedness and Planning of Academic Emergency Departments in India During the COVID-19 Pandemic: A Multicentric Survey

**DOI:** 10.1017/dmp.2021.73

**Published:** 2021-03-10

**Authors:** Vivek Gopinathan, Sanjan Asanaru Kunju, Vimal Krishnan S, Freston Marc Sirur, Jayaraj Mymbilly Balakrishnan

**Affiliations:** 1 Department of Emergency Medicine, Kasturba Medical College, Manipal, Manipal Academy of Higher Education, Manipal, Karnataka, India; 2 Department of Emergency Medicine, Kasturba Medical College, Manipal, Manipal Academy of Higher Education, Manipal, Karnataka, India; 3 Department of Emergency Medicine, Kasturba Medical College, Manipal, Manipal Academy of Higher Education, Manipal, Karnataka, India; 4 Department of Emergency Medicine, Kasturba Medical College, Manipal, Manipal Academy of Higher Education, Manipal, Karnataka, India; 5 Department of Emergency Medicine, Kasturba Medical College, Manipal, Manipal Academy of Higher Education, Manipal, Karnataka, India

**Keywords:** emergency departments, COVID-19 pandemic, disaster preparedness

## Abstract

**Objective:**

Emergency medicine being a young specialty in India, we aimed to assess the level of disaster preparedness and planning strategies among various academic emergency departments (EDs) across India during the coronavirus disease 2019 (COVID-19) pandemic.

**Methods:**

A cross-sectional multicentric survey was developed and disseminated online to various academic EDs in India and followed up over a period of 8 wk. All results were analyzed using descriptive statistics.

**Results:**

Twenty-eight academic emergency medicine departments responded to the study. Compared with pre-COVID period, COVID-19 pandemic has led to 90% of centers developing separate triage system with dedicated care areas for COVID suspected/infected in 78.6% centers with nearly 70% using separate transportation pathways. Strategizing and executing the Institutional COVID-19 treatment protocol in 80% institutes were done by emergency physicians. Training exercises for airway management and personal protective equipment (PPE) use were seen in 93% and 80% centers, respectively. Marked variation in recommended PPE use was observed across EDs in India.

**Conclusions:**

Our study highlights the high variance in the level of preparedness response among various EDs across India during the pandemic. Preparedness for different EDs across India needs to be individually assessed and planned according to the needs and resources available.

Hospitals and other health-care facilities play a critical role in national and local responses to emergencies during infectious disease outbreaks like coronavirus disease 2019 (COVID-19). COVID-19 pandemic has impacted nations across the world with a devastating toll of 60 million cases worldwide with more than 1.42 million deaths as of writing this on November 26, 2020.^1^ The coronavirus is changing all of our lives and has led to a significant public health crisis with an impact on multiple domains and had disrupted lives, economies, and societies. Nevertheless, the hospitals are expected to function even during the disaster with a massive patient surge catering to a safe environment for personnel in addition to providing essential medical care to the affected people.^2^ Within the health-care system, the emergency department (ED) being the main entry point of patients to hospital care, it is of utmost importance that it should be optimally prepared to manage high-risk COVID-19 patients from triage to final disposition.^3^

Preparedness must achieve orderly transitions from response through to sustained recovery from the impact of likely or current hazard events or conditions with activities such as contingency planning, stockpiling of equipment and supplies, the development of arrangements for coordination and associated training and drill exercises.^4^ The hospitals will have to face numerous challenges confronting the massive influx of patients suffering from emerging infectious diseases, and an emergency disaster management program is essential for hospital preparedness to deal with all probable hazards.^5,6^ India being the second-most populous country in the world, preparedness is imperative to respond to the situation with increasing severity of outbreaks, especially in the densely populated states. While specific preparedness activities differ between types of health-care institutions and threat phases, developing a uniform enhanced preparedness system helps to mitigate the disaster.^7-10^ Proper plan about the flow pattern of the patient with treatment protocol is required to provide appropriate care to patients with suspected COVID-19 while avoiding delay in care of non-COVID-19 patients.^11^ In addition, it is paramount to ensure that health-care workers (HCWs) are adequately protected.^12^ Through this study, we aim to understand the preparedness and response of various EDs in the country during the COVID-19 pandemic, identify the variability and, thus, facilitate further research for the formation of a policy/plan that can be used for disaster preparedness in the future.

## Methods

### Study Setting

We conducted a cross-sectional multicentric survey. The survey draft underwent multiple rounds of review by the research team. The study protocol was reviewed and approved by the Institutional Research Board and Institutional Ethics Committee (Registration No. ECR/146/Inst/KA/2013/RR-19) (DHR Registration No.EC/NEW/INST/2019/374) IEC: 297/202. The survey was distributed to the country’s major EDs by means of electronic mail. The participation of the individual institute is pure voluntarily with no personal benefits. An ED consultant with a minimum 2-y work experience consenting to be a part of the survey was the nodal point at each institute. After mailing the survey questionnaire, a period of 4 wk was given for the participants to complete it. A maximum of 2 electronic reminder emails were sent. The permission to access the survey questionnaire is given only through their email address to avoid the duplication of the response from an institute. The questionnaire contains an initial part explaining the purpose, objective, and consent form. The access to the questionnaire will be granted only after consenting to the survey.

The survey was open on June 10, 2020, and completed by August 20, 2020. Survey responses were collected and retrieved through Google Forms, a well-known online data collection system. The ED survey completed by each participating center focused on the organizational and operational aspects of pandemic preparedness taken by the EDs, including contingency plans, surge capacity, and the use of personal protective equipment (PPE), care pathways, and safety of health-care professionals.

### Primary Data Analysis

Descriptive statistics were used to analyze the data. Median and interquartile range (IQR) was reported for continuous variables. Categorical variables were assessed and described as frequency and percentage. Data analyses were performed using the Statistical Package for the Social Sciences for Windows, Version 23.0 (SPSS Inc. - IBM Corp., Armonk, NY).

## Results

### Pre-COVID

A total of 28 institutes had participated in this study after obtaining consent from their respective institutions. Baseline characteristics and resources of each of the participating institutes are included in Table 1. A total of 89.3% institutes had an existing disaster preparedness committee among which 85.7% had a representation from emergency medicine (EM) as a part of their hospital disaster preparedness plan.

**Table 1.** Characteristics of the institutes before the COVID-19 pandemic

Characteristics	Median	Q1	Q3	IQR
No. of consultants	4	2	5	3
No. of residents	6	3	9	6
No. of nurses	34	20	42.5	22.5
No. of nurses work per shift	7	4.5	14.5	10
Total beds in ED	30	16.5	39.25	22.75
Resuscitation beds in ED	6	3.5	9.25	5.75
No. of ICU beds attached to ED	10	6	20	14
Ventilators in ED	4	3	10	7
Average no. of patients/day	80	50	120	70
ICU beds in hospital	75	39	105	66

Abbreviations: Q1, quartile 1; Q3, quartile 3.

### COVID Period

#### Patient Flow Pattern

**Figure 1.**
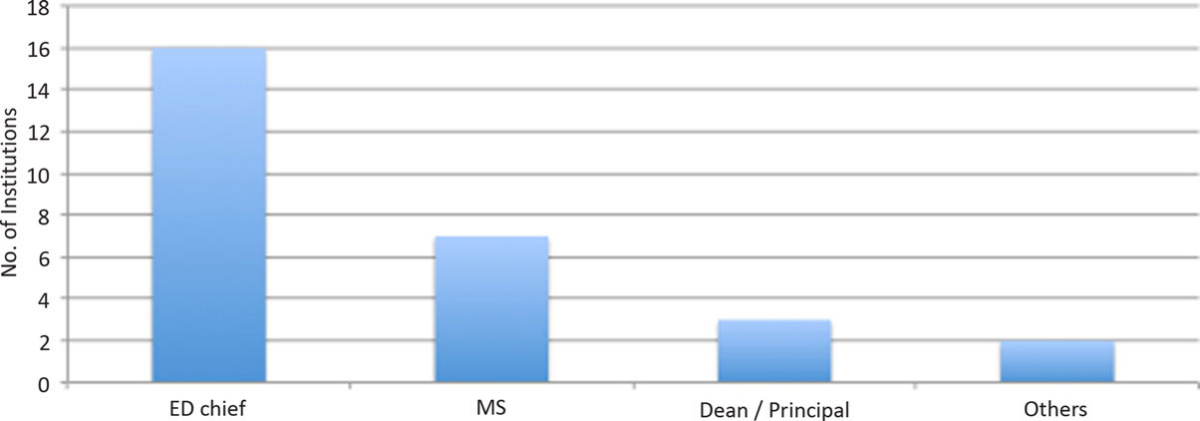
Nodal officer directing patient flow during COVID-19 pandemic. Abbreviations: COVID-19, coronavirus disease 2019; ED Chief, Emergency department chief; MS, Medical superintendent.

Major changes in the patient flow at the ED were evident. Health-care institutions to reduce or prevent exposure to hazards had mandated the policy regarding patient flow. The administrative officer responsible for determining and executing these policies was the ED chief in 57% of the institutes (Figure 1). The objective of our study was to focus on the preparedness of EDs during the COVID-19 pandemic and hence comprehensive patient flow patterns in the rest of the hospital (excluding ED) were not extensively studied.

#### Triage

**Figure 2.**
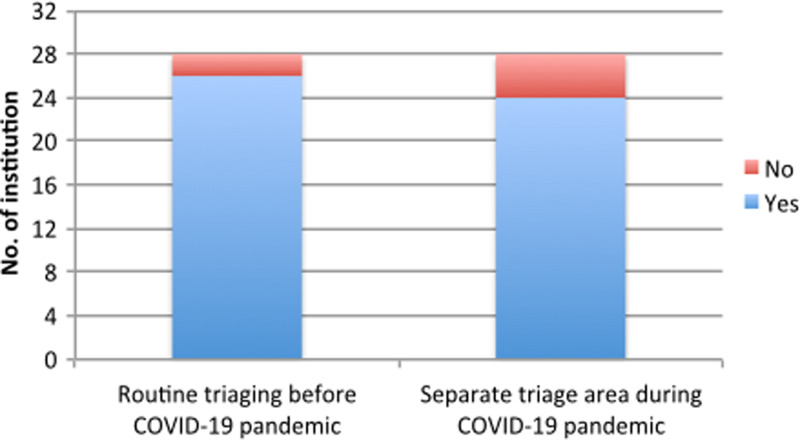
Triaging and COVID-19 pandemic. Abbreviations: COVID-19, coronavirus disease 2019.

Even before the pandemic, 90% or more of the participating centers had a proper triaging system for the patients. However, during the pandemic, 10% or more did not have separate triaging to deal with the surge of suspected or confirmed COVID-19 cases (Figure 2).

### Patient Care Dynamics

#### Noncritical COVID Patient/Suspect Care Pathway

**Figure 3.**
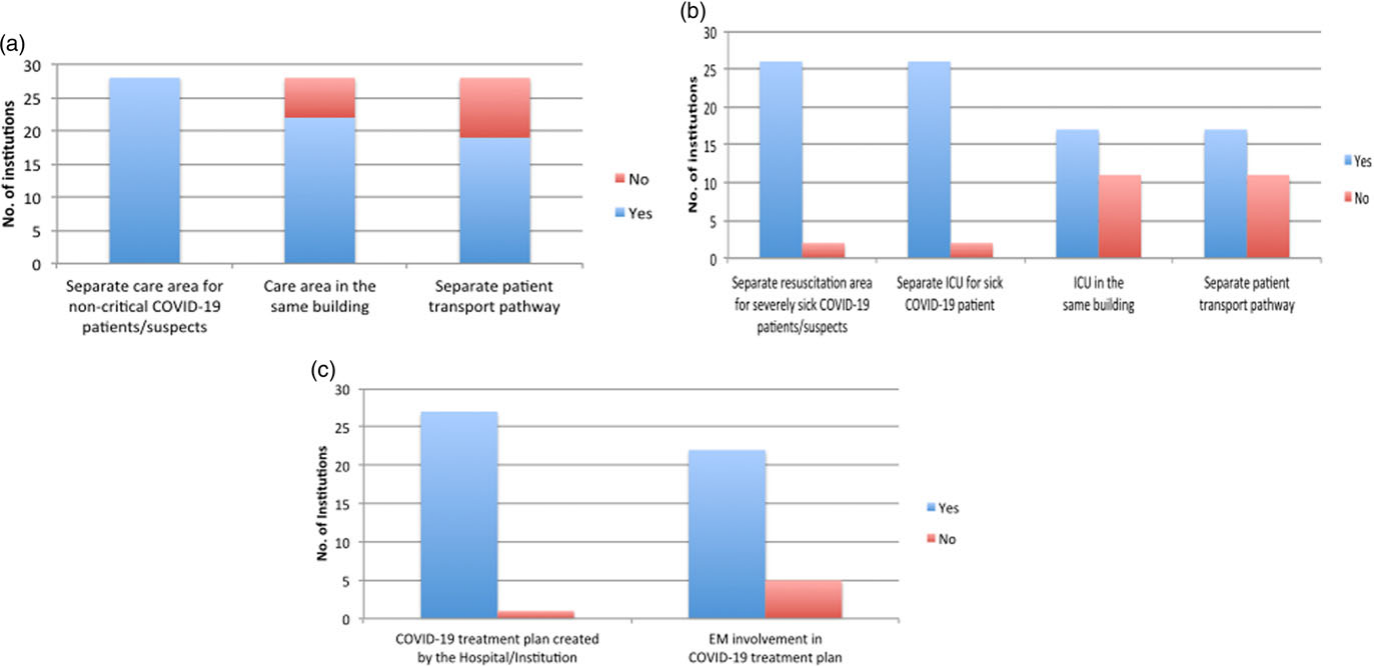
Patient care dynamics. (a) Separate care area and transport pathway for non critically ill COVID-19 patients. (b) Separate care area and transport pathway for non critically ill COVID-19 patients. (c) COVID-19 treatment plan by institute and involvement of EM. Abbreviations: COVID-19, coronavirus disease 2019; ICU, Intensive care unit; EM, Emergency Medicine.

All centers had separate areas for receiving and treating the noncritical patients during the pandemic (Figure 3a). Nearly 78.6% (*n* = 22) centers had a dedicated care area for the COVID patients in the same building. Safe transportation of critically ill and suspected COVID patients are always challenging, and nearly 70% of the centers had used separate pathways for the transportation of these patients to minimize nosocomial spread to other patients and HCWs (Figure 3a).

#### Critical COVID Patient/Suspect Care Pathway

Approximately less than 10% did not have a contingency plan of separate resuscitation areas for severely sick COVID-19 suspects/patients. Intensive care unit (ICU) capacity for COVID-19 patients had been expanded in most centers (90%) where nearly 60% had it in the same building with a separate pathway for the safe transportation of the patient (Figure 3b).

#### Treatment Plan for COVID Patients

Except for 1 center in India, all institutes had developed definite treatment plans for safe participation in inpatient care. Being the frontline HCWs who faced all the new challenges during this pandemic, there was active participation by EM specialists in almost 80% institutes for strategizing and executing the treatment protocols of COVID-19 management (Figure 3c).

### Training

**Figure 4.**
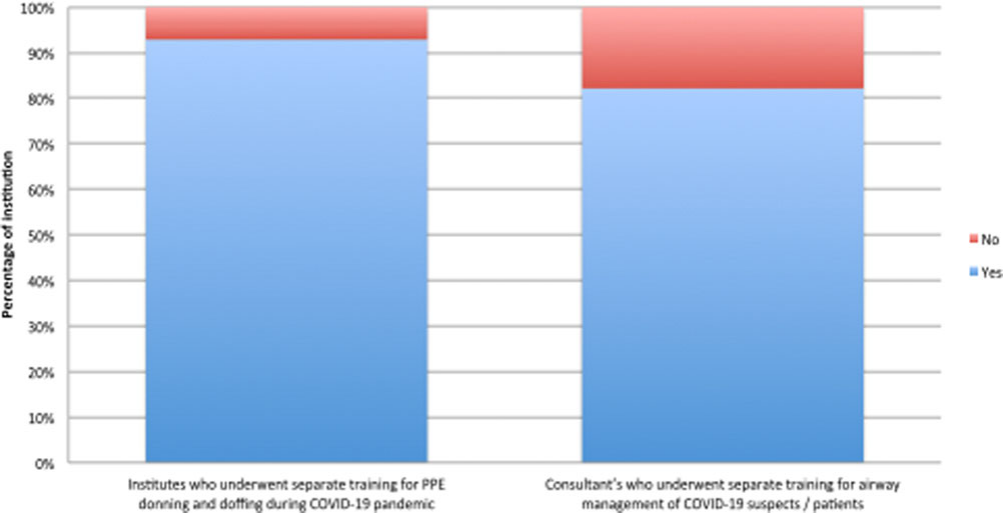
PPE use and Airway management training during the COVID-19 pandemic. Abbreviations: COVID-19, coronavirus disease 2019; PPE, Personal protective equipment.

Approximately 93% of the institutes initiated training programs for donning and doffing for HCWs during the COVID-19 pandemic. We have also noted that nearly 80% of EM consultants underwent training for airway management at their respective institutes (Figure 4).

### PPE Use and COVID-19 Pandemic

**Figure 5.**
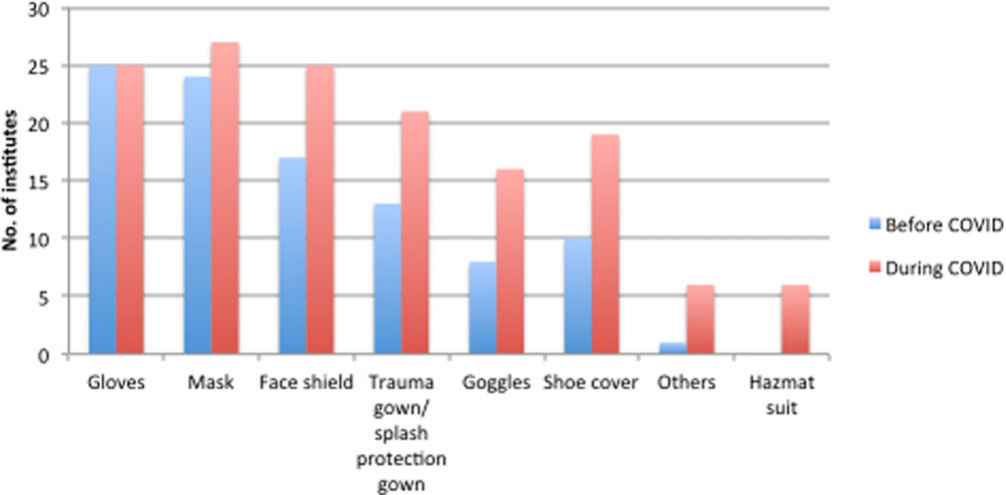
PPE use before and after the COVID-19 pandemic.

The distribution showed the variation in the availability of PPE before and during the COVID-19 pandemic (Figure 5). Although all health-care personnel were trained on the indications and proper use of PPE, marked variation was observed in the recommended use of PPE to be worn in various institutes during and before the COVID-19 pandemic (Table 2). Furthermore, eye protection is essential as inoculation of the conjunctival surface can also result in COVID-19 infection.^13^ To prevent the transmission through conjunctiva, there had been a markedly increased use of the face shield (from 60% to 90%), as well as trauma gown/splash (from 46% to 75%), goggles (from 28% to 57%) during the COVID-19 pandemic. Only 20% of institutes had implemented the hazmat suit for caring for COVID-19 patients according to our survey.

**Table 2.** PPE use before and during the COVID pandemic

PPE before COVID-19	No. of institutes	PPE during COVID-19	No. of institutes
G, M, FS, TG/SPG, Go,SC	5	G, M/N95M, FS, TG/SPG, Go, SC, HS, Others	3
G, M, FS, TG/SPG, SC	4	G, M/N95M, FS, TG/SPG, Go, SC, HS	2
G, M, TG/SPG, Go, SC	1	G, M/N95M, FS, TG/SPG, Go, SC, others	1
G, M, FS, TG/SPG	2	G, M/N95M, FS, TG/SPG, Go, SC	8
G, M, FS, TG/SPG	1	G, M/N95M, FS, TG/SPG, SC, HS	1
G, M, FS, Go	2	G, M/N95M, FS, TG/SPG, SC,others	1
G, M, FS	4	G, M/N95M, FS, TG/SPG, Go	1
G, M	3	G, M/N95M, FS, TG/SPG, SC	1
G	3	G, M/N95M, FS, Go, SC	1
M	2	G, M/N95M, FS, TG/SPG	3
Others	1	G, M/N95M, FS, SC	1
		G, M/N95M, FS	2
		M/N95M	2
		Others	1

Abbreviations: G, gloves; M/N95M, mask/N95 mask; FS, face shield; TG/SPG, trauma gown/splash protection gown; Go, goggles; SC, shoe covers; HS, hazmat suit.

## Discussion

Newer and relatively unknown infectious diseases causing pandemics constitute a significant concern in the current global scenario. Communicable diseases are expected to remain a significant global public health concern in the coming decades.^14^ India is the second-most populous country on earth with 1.36 billion people. So, the recent outbreak of COVID-19 in India is a global health issue. Currently, India stands at second position with almost 9 million infected patients.^1^

### Emergency Medicine and COVID-19 Pandemic in India

EM is still a nascent specialty, and its development has been protracted. The first Government of India approved, Medical Council of India (MCI) recognized academic EM residency program was initiated in 2009. There are currently 41 medical colleges and close to 200 seats of EM in India of which 28 institutes consented and responded to the national survey conducted. The reason for choosing academic EM than random EDs was due to the assumption that there would be more structured protocols and policies in line with the current World Health Organization (WHO) guidelines.^15^

Adequate human resource is 1 of the cornerstones of optimizing ED performance. Even during the pandemic, most of the facilities had only a median number of 4 EM consultants and an average of 6 residents with some of the institutes having no residents at all. The recent doctor to population ratio in India is at a staggering 1:1596. This is way below the international standards.^16^ Deaths among HCWs involved in COVID-19 care in India stands at 665.^17^ However, the mortality rates of HCWs in India are almost comparable to that found in other parts of the world.^17,18^ The average number of nurses in an ED varied among different programs, with 34/d being the median. The usual shift pattern adopted for COVID-19 care was 8 h compared with the 6-h shift pattern observed in many countries.^19^ Changes in duty pattern/work schedule may contribute to psychological stress for the frontline HCW and lower the patient care quality and safety.^20,21^

The overwhelming number of COVID-19 patients demands the reorganization of the health-care system and adapted changes to maximize resources to reduce patient crowding and reduce potential nosocomial COVID-19 spread. The average number of beds in ED was found to be 30 with 6 beds set apart for resuscitation, and as much as 60.7% of the EDs had an ICU attached to it for early critical care after resuscitation with an average of 10 beds.

A good indication of the disaster preparedness was highlighted during COVID-19 pandemic when a separate preparedness and management plan was formulated in 96.4% of centers where the EM department was involved in the plan creation 81.5% of the time.^22^

### PPE Use During COVID-19 Pandemic

PPE ranging from simple mask to hazmat suit (Level C PPE) has become an important part of HCW’s, life especially in the context of community transmission, as asymptomatic patients may also be carriers of the virus.^23^ PPE remained a concern during the initial stages; however, it had evolved from general to specific PPE to be worn over time.^24^ We also noted that some centers were using only minimal protection (mask only/gloves only) before the pandemic, while there was a definitive improvement in the use of PPE during the COVID-19 pandemic. The use of PPE, however, was very heterogeneous in EDs across the country. This is in comparison with the European EDs showing varied use of PPE among departments during the pandemic.^25^ The PPE use pattern is reflective of the planning and preparedness of the EDs during disasters.

### Triage and Patient Care Areas During COVID-19 Pandemic

Even though the majority of the EDs in India had a routine triaging system, the COVID-19 pandemic has led to the opening up of separate triage systems as a part of the emergency preparedness plan. According to our survey, in many institutes, this COVID-19 triage area was in the same building. This was in accordance with studies showing similar results.^26^

COVID-19 had produced not only overcrowding of health-care facilities by patients with severe acute respiratory illness but also a change in the care area and flow pattern of the patient.^27^ We also noted that there was a surge in resuscitation beds with a separate patient flow pattern for critically ill patients during the current pandemic in all the centers. The majority centers (92.9%) ensured a separate patient transport pathway for COVID-19 suspected/positive patients of which 65.4% had a dedicated ICU in the same building. However, hospitals are innovating their processes, especially the ICU facilities to separate COVID-19 patients from non-COVID-19 patients.

### Training During the Pandemic

Regular training on PPE use and airway management of critically ill COVID-19 patients were valuable to improve the performance of frontline HCWs. Donning and Doffing training with concurrent involvement in simulation exercises targeting resuscitation of COVID-19 patients was 1 of the strategies used to mitigate the risk of transmission to health-care professionals and to patients.^28^

Airway management in COVID-19 patients was identified as a challenging issue in the majority of centers, and we observed that 82% of EM consultants underwent separate airway training exercises during the COVID-19 pandemic.^29^ This highlights the importance of simulation exercises and targeted training for the current pandemic.

### Challenges in Non-COVID Patient Care During the COVID-19 Pandemic

There was almost a 50% decline in cases of non-COVID emergencies during the pandemic. This was more evident during the lockdown period in India.^30^ There were various other challenges during the pandemic, such as difficulty in reaching the hospital due to issues with transportation, shortage of resources, and additional time spent for donning and doffing.^31^

Burnout among frontline HCWs may have impacted the quality of care delivered during the COVID-19 pandemic as revealed by the study published earlier from our center.^20^ There is a need for incorporating wellness strategies in preparedness planning during the pandemic.

### Limitations

The study was limited to academic EM departments in India, which cannot be extrapolated to the entire country. Academic EM in India is still in its infancy but does not justify the number of centers who have been part of the survey. The pandemic may have limited the centers agreeing to be part of the survey. Disaster preparedness in academic EDs in India are not extensively studied earlier, and the lack of adequate previous data for reference may have influenced the overall impact of the study.

## Conclusion

Overall, our study findings had shown high variance in the level of preparedness among various EDs across India in response to the COVID-19 pandemic. The study reiterates that a blanket plan for preparedness for all EDs might not be ideal but rather an individualized strategy understanding the baseline needs and working with a targeted plan for each state, would be the ideal strategy. Capacity building in emergency care services is the need of the hour and the new guidelines from the National Medical Commission mandates an ED with adequate resources.^32^ New government policies to increase the bed strength and to provide patient care at a cost-effective price for the patient should be implemented. All HCWs must be guaranteed a minimum necessary PPE for routine use and have provisions for hiking up during disaster situations. More research is needed for a better understanding of the dynamics of preparedness and planning in India during a pandemic, as there is a paucity of data on the same.
